# LOAD-intensity and time-under-tension of exercises for men who have Achilles tendinopathy (the LOADIT trial): a randomised feasibility trial

**DOI:** 10.1186/s13102-021-00279-z

**Published:** 2021-05-25

**Authors:** Fatmah Hasani, Terry Haines, Shannon E. Munteanu, Peter Schoch, Bill Vicenzino, Peter Malliaras

**Affiliations:** 1 Physiotherapy Department, School of Primary and Allied Health Care, Monash University, Frankston, Melbourne, Victoria 3199 Australia; 2 Physiotherapy Department, Security Forces Hospital, Riyadh, 11481 Kingdom of Saudi Arabia; 3 School of Primary and Allied Health Care, Faculty of Medicine, Nursing, and Health Sciences, Monash University, Frankston, Victoria 3199 Australia; 4 Discipline of Podiatry, School of Allied Health, Human Services and Sport, College of Science, Health and Engineering, La Trobe University, Melbourne, Victoria 3086 Australia; 5 La Trobe Sport and Exercise Medicine Research Centre, School of Allied Health, Human Services and Sport, College of Science, Health and Engineering, La Trobe University, Melbourne, Victoria 3086 Australia; 6 McKenzie Institute Australia, Beaumaris, Victoria 3193 Australia; 7 McKenzie Institute International, Raumati Beach, 5032 New Zealand; 8 Physiotherapy Department, Barwon Health, Geelong, 3220 Australia; 9 Sports Injuries Rehabilitation and Prevention for Health Research Unit, The University of Queensland, School of Health and Rehabilitation Sciences, Brisbane, Queensland Australia

**Keywords:** Achilles tendinopathy, Rehabilitation, Exercise parameters

## Abstract

**Background:**

One potential reason for disparate outcomes of exercise for Achilles tendinopathy is poor knowledge about whether exercise parameters (i.e. different exercise doses) influence outcome. Whether parameters that are important for tendon adaptation influence clinical outcomes in Achilles tendinopathy has not been investigated. Therefore, this research aimed to assess the feasibility of conducting a fully powered randomised trial to investigate the efficacy of different load-intensity and time-under-tension exercise parameters for Achilles tendinopathy.

**Methods:**

A factorial four-arm, randomised trial. Forty-eight male participants (18–70 years old) with mid-portion Achilles tendinopathy (≥ 3 months) were recruited. Participants were randomly allocated to high (6 repetition maximum) or low intensity (18 repetition maximum) exercise, performed with either high (6 s per cycle) or low (2 s per cycle) time-under-tension. Participants performed 12-weeks of standing and seated calf raise exercises three times per week in a gym setting using a Smith machine. One session per week was supervised (via videoconference). Primary feasibility outcomes (recruitment and retention rate, exercise adherence and fidelity [i.e. time-under-tension, volume, load intensity], incidence of adverse events, health care use and productivity cost) were collected weekly. Means and standard deviations were determined for parametric data, medians and interquartile range for non-parametric continuous data, and frequency counts for discrete data.

**Results:**

Total recruitment (76%) and retention (90%) rates were high. Exercise adherence ranged from 45 to 63% and fidelity ranged from 8 to 83% across the groups. Thirty-one participants reported 64 adverse events over the 3 months. Twenty-one participants (70%) reported mild events. Participants reported reduced presenteeism more than absenteeism.

**Conclusions:**

A fully powered trial is feasible. The proposed trial design and interventions demonstrated acceptable recruitment and retention rates and safety profile. However, exercise fidelity and adherence to the gym-based intervention was not acceptable. Strategies to improve intervention adherence and fidelity should be considered in future trials.

**Trial registration:**

Australian New Zealand Clinical Trials Registry, ACTRN12618001315202. Registered retrospectively on August 6th, 2018.

**Supplementary Information:**

The online version contains supplementary material available at 10.1186/s13102-021-00279-z.

## Clinical messages

- Conducting a fully powered randomised trial to determine the efficacy of different load-intensity and time-under-tension exercise parameters for Achilles tendinopathy is feasible.
- Strategies designed to improve exercise adherence and fidelity are necessary prior to progressing to a fully powered trial.
- The findings provide important preliminary information regarding treatment effect sizes of the interventions described.

## Background

Mid-portion Achilles tendinopathy is a common musculoskeletal condition characterised by localised Achilles tendon load-related pain and dysfunction [1]. The pain mechanisms in tendinopathy are not clear but are thought to involve local nociception [2]. The etiology of tendinopathy is complex and multifactorial [3], but imbalance between the load demands placed on the tendon and its capacity to remodel is considered a major factor [4]. Both athletes and sedentary people can be affected, and many suffer profound and longstanding impairment of activities such as walking and running [5, 6].

Although calf loading exercise is promoted as an evidence-based treatment, there is no clear guidance from the research about which exercise approach is optimal [7–9]. This may be because; i) the efficacy of different exercise parameters have not been adequately investigated; ii) exercise approaches are poorly reported so hard to compare; iii) exercise adherence is either not reported or reported with untrustworthy measures; and iv) the specific exercise approach may not matter as much as other factors (e.g. psychological factors such as conceptualisation of pain) [6, 8, 10, 11].

A sensible first step and the focus of the trial reported here was to develop evidence for whether exercise parameters influence outcomes in Achilles tendinopathy. Many parameters can be influenced in exercise prescription to have specific effects on the musculotendinous unit, including load-intensity (e.g. repetition maximum [RM], maximal voluntary contraction), volume (repetitions and sets), and time under tension per contraction (repetition duration) [12]. A systematic review investigating exercise response (i.e. adaptative outcomes such as tendon stiffness) in healthy Achilles and patellar tendons concluded that load-intensity is a key determinant of tendon tissue adaptation to load, and the type of contraction (e.g. eccentric versus concentric) did not influence adaptation [13]. Load-intensity results in greater tendon tissue strain which deforms tendon cells and triggers anabolic cell signalling [14]. There is also evidence that longer duration contractions at the same intensity result in greater Achilles tendon adaptation [15], most likely because there is time-dependent transmission of external load to the tendon cytoskeleton and cells. Whether parameters that are important for tendon adaptation such as load-intensity and duration of contraction (or time-under-tension) influence clinical outcomes in Achilles tendinopathy has not been investigated.

The primary aim of this study was to determine the feasibility of conducting a fully powered randomised trial to determine the efficacy of different load-intensity and time-under-tension exercise parameters for Achilles tendinopathy. Maximising and monitoring exercise adherence and fidelity are integral to the validity of conclusions that can ultimately be made from such a fully powered trial, so we chose to use regular telehealth to efficiently monitor (and encourage) these factors. The key outcomes were (i) rate of participant recruitment, conversion, and retention, (ii) ability to perform the interventions per-protocol (adequate exercise fidelity and adherence based on weekly videoconference assessment), (iii) incidence and type of adverse events, (iv) use of rescue medication and co-interventions, and (v) feasibility of future economic evaluation. The secondary aim was to provide estimates of the variability of key outcomes. Outcomes were informed by a recent international consensus on core outcomes for tendinopathy [16] and included pain and disability, satisfaction, physical activity, health-related quality of life, psychological measures and plantarflexor function. In terms of plantarflexor function capacity we assessed maximal voluntary contraction, rate of torque development and force steadiness because these may be impaired among individuals with Achilles tendinopathy [17, 18].

## Methods

### Design

**Fig. 1:**
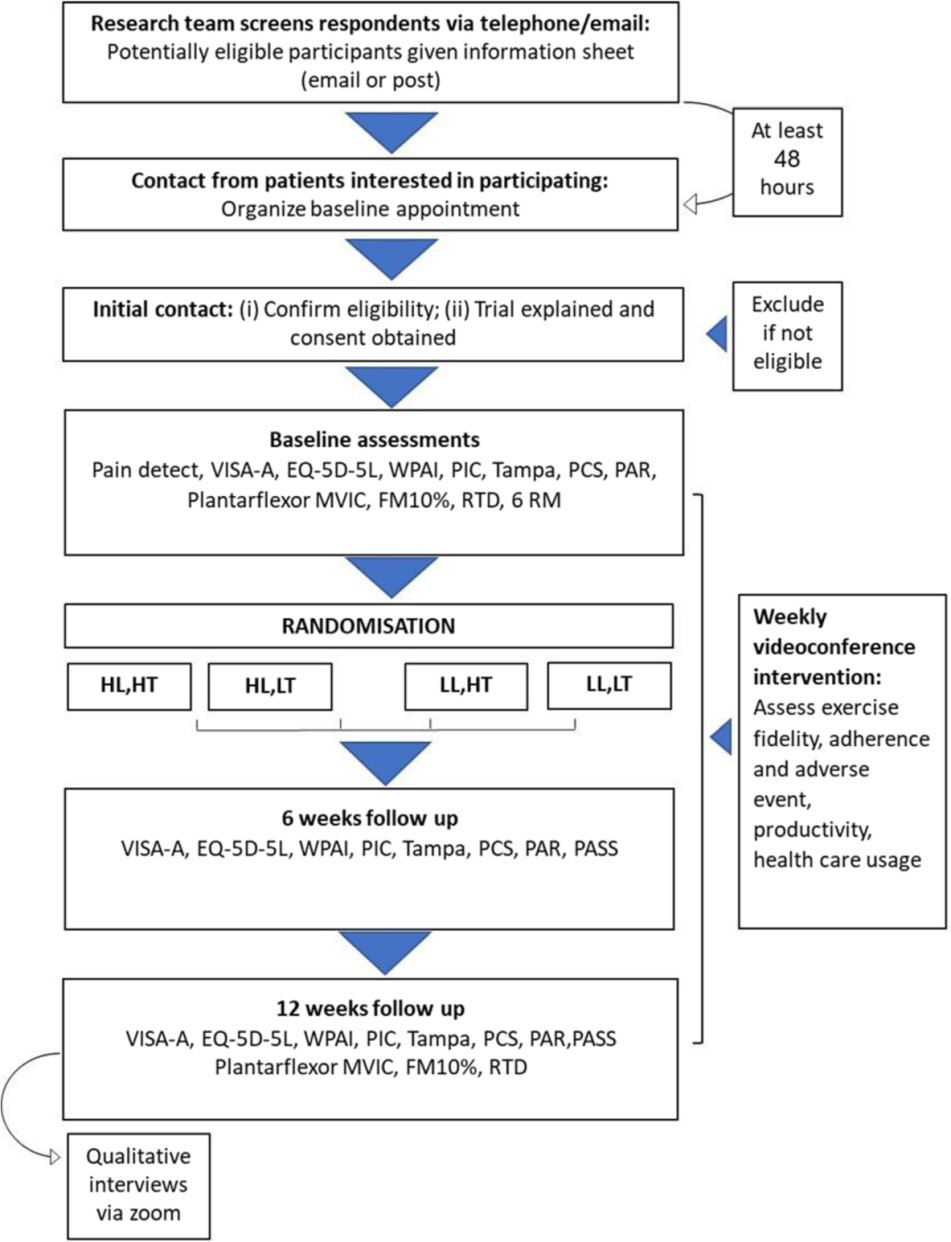
Trial profile. Abbreviation: HL, High load; HT, High time-under-tension; LL, Low load; LT, Low time-under-tension; VISA-A, Victorian Institute of Sports Assessment – Achilles; EQ-5D-5L, Health-related Quality of Life; WPAI, Work Productivity and Activity Impairment; PIC, Patient Impression of Change; PCS, Pain Catastrophising Scale; PAR, Physical Activity Recall questionnaire; PASS, Patient-Acceptable Symptom State instrument for satisfaction; MVIC, Maximal Voluntary Isometric Contraction; FM10%, Force match at 10%; RTD, Rate of Torque Development; RM, repetition maximum

LOADIT (LOAD- Intensity and Time-under-tension) is a four-arm, factorial randomised pilot trial. The two factors, each with two levels, are load-intensity (determined by RM, the maximum mass that can be lifted for a given number of repetitions) and time-under-tension (determined by seconds). Participants were randomly allocated into one of four groups: 6 RM with 2 s repetitions; 6 RM with 6 s repetitions; 18 RM with 2 s repetitions; or 18 RM with 6 s repetitions group (Fig. 1). The methods are described in the published protocol [19]. The study was reported in accordance with the CONSORT extension for randomised pilot and feasibility trials [20] and the TIDieR guide [21]. The protocol was registered (August 2018; ACTRN 12618001315202. The trial was approved by the Human Research Ethics Committee at Monash University (ethics number 2018–1366-20,711).

### Study population

Participants were included if they were male, aged between 18 to 70 years with a history of mid-portion Achilles tendon pain in either or both legs for ≥12 weeks, and scored ≤75 on the Victorian Institute of Sports Assessment – Achilles questionnaire (VISA-A). Men were the focus because of evidence that tendon adaptation to exercise (and therefore possibly clinical outcomes) may be confounded by sex [22]. The clinical diagnosis was based on the clinical presentation, physical examination and ultrasound imaging done by practicing physiotherapist (FH). Exclusion included a history of Achilles tendon rupture, surgery in the symptomatic lower limb(s) or any health conditions that may interfere with the execution of the exercise interventions. Participants were also excluded if they had had an injection or received strength exercise treatment for their Achilles tendon pain within the last 3 months.

### Recruitment and setting

Participants were recruited via social media (i.e. Facebook, Twitter), and by posting study information on relevant internet websites (e.g. sports clubs and forums), as well as referral from health professionals in Melbourne. All screening assessments and data collection were conducted at a single centre (Monash University, Melbourne, Victoria, Australia) between July 2018 and May 2019. Responders to the advertisements were asked to provide their contact number. They were contacted and screened via the telephone by research assistants who also provided trial information. Those who confirmed interest in the study (either at the time of the telephone call or by re-initiating contact with the researcher after the initial call) were invited to attend a screening visit to confirm their eligibility. All participants provided oral and written informed consent before joining the trial.

### Randomisation and blinding

A randomisation sequence with permuted blocks of variable size was created using an online randomisation service (Sealed Envelope Ltd., London) and then concealed in opaque sealed envelopes by a researcher who was not in contact with participants (TH). The treating physiotherapists and participants were not blinded. Each participant received a scripted explanation of the trial which included that there is uncertainty about whether any of the exercise interventions would be superior. The participants were assigned to intervention by the same investigator (PM). The investigator (FH) who administered and collected the secondary outcomes and the statistician were blinded to group allocation.

### Exercise interventions

Participants performed four sets of unilateral standing and seated isotonic calf raise exercise to load the ankle plantarflexor complex (both sides, one leg at a time), three times per week for 12 weeks, with standardised rest times (90 s between sets). The 12-week endpoint was chosen because it has been shown to be a sufficient timeframe for exercise to have a clinically meaningful effect on Achilles tendinopathy pain and function [23]. Both isotonic exercises were performed in a Smith machine at a local gymnasium (gym membership was provided free of charge to participants, if required). A practicing physiotherapist monitored one session per week (via videoconferencing software [Zoom®]) and provided exercise progression and technique feedback. In each session, the physiotherapists rated the exercise fidelity criteria as achieved or not achieved per our protocol i.e. prescribed number of repetitions, sets, RM and speed of contraction [19]. During the weekly telehealth session, exercise adherence and fidelity (prior to providing feedback), adverse events, use of co-interventions, and productivity data were collected. Participants were taught calf raise exercise technique [19] using parameters specific to their group allocation (Table 1) and the exercises were externally paced using a metronome (via smartphone application). Participants were instructed to exercise to volitional failure and how to progress and regress exercise based on difficulty with the exercise and pain experienced. The instruction to participants was that they should feel that they cannot do any more repetitions when they complete the set. A range of ± one repetition and pain with load < 5 out of 10 on numerical rating scale is allowed. After the baseline testing of the repetition maximum, exercise intensity was adjusted down by 10% in all groups to reduce the risk of muscle soreness related to commencement of unaccustomed exercise. Further exercise details are provided in additional file 1 and the protocol [19].

**Table 1:** Calf exercise dosage for each group

	High Load		Low Load	
	with High TUT (Group 1)	with Low TUT (Group 2)	with High TUT (Group 3)	with Low TUT (Group 4)
**Load intensity**	6 RM	6 RM	18 RM	18 RM
**Repetition**	6	6	18	18
**Sets**	4	4	4	4
**Contraction time/rep**	3 s concentric 3 s eccentric	1 s concentric 1 s eccentric	3 s concentric 3 s eccentric	1 s concentric 1 s eccentric
**Rest in between reps**	No	No	No	No
**Contraction time per set**	36 s	12 s	108 s	36 s
**Total contraction time**	288 s	96 s	864 s	288 s
**Volitional muscular failure**	Yes	Yes	Yes	Yes
**Range of motion**	0° to 15° DF 0° to 50° PF	0° to 15° DF 0° to 50° PF	0° to 15° DF 0° to 50° PF	0° to 15° DF 0° to 50° PF

*Abbreviations*: *TUT* time-under-tension, *RM* repetition maximum, *TUT* time-under-tension, *DF* dorsiflexion, *PF* planter flexion, *S* seconds

Participants were provided with education related to tendon pain mechanisms and acceptable levels of pain during exercise and activity. Participants were advised to consume up to four g/day pain-relieving medication [24] (i.e. paracetamol), if required. Participants received advice to gradually increase walking, running and sports activity if Achilles tendon pain during these activities was not beyond level 5 out of 10 on an 11-point numerical pain rating scale (NPRS) 0 = no pain, 10 = worst pain imaginable) [25]. They were also advised it was acceptable for pain after sport activities to temporarily increase as long as it returned to baseline levels on a tendon loading test such as single-leg submaximal hop or single leg calf raise, within approximately 24 h.

### Outcome measures

#### Primary outcomes

##### Rate of conversion, recruitment, and retention

The conversion rate was the proportion of people who consented divided by those who met the criteria. The recruitment rate was the number of participants recruited per month. Retention was the proportion of recruited participants who completed the 12-week outcome assessment. The conversion and retention success criteria were ≥ 20 and 80% respectively.

##### Exercise adherence and fidelity

Adherence was the proportion of prescribed exercise sessions completed or attempted of the total of 36 and was expressed as the frequency and percentage. Fidelity was the proportion of weeks (out of 12) where participants achieved adequate fidelity in each parameter i.e. tempo per contraction [time-under-tension], volume [repetitions and sets] and intensity [failure ± one repetition]. It was also expressed as frequency and percentage. Achieving ≥66% in adherence and fidelity was deemed a success criterion for the trial. We are not aware of any established minimum level of exercise fidelity for a trial like this but reasoned that exercise fidelity that was below this threshold of 66% (whether across all groups or only in a proportion of groups) may impact on the effectiveness of the protocol exercise parameters and limit the certainty of any conclusions.

##### Incidence of adverse events

An adverse event was defined as any unintended symptom associated with the study which may or may not be related to the intervention [26]. The frequency (number of participants and number of cases), nature (e.g. sprained ankle, a muscle tear or tendon pain worsening), and severity (mild [< 48 h], moderate [up to 7 days], or severe [> 7 days or requiring medical attention]) were recorded at the weekly telehealth session. Individuals experiencing adverse events were managed by the research team or triaged to an appropriate medical facility.

##### Use of co-interventions

The frequency of the use of paracetamol medication and other co-interventions was recorded.

##### Feasibility of future economic evaluation

These costs were divided into the following:

- The direct intervention costs: This included the gym membership (estimated 15 AUD per week), physiotherapy treatment and participant screening time (estimated rate 150 AUD per hour). Other direct cost for co-interventions were calculated based on number of days the co-intervention × market price or by estimating the once off cost of the health product.
- The indirect cost or productivity cost included the absenteeism (time loss from work due to Achilles tendinopathy) and presenteeism (productivity loss while at work due to Achilles tendinopathy) were assessed using the Health-related work productivity questionnaire (WPAI) [27]. Absenteeism cost was calculated by multiplying this percentage score × the average wage rate in Australia (estimated at 45 AUD per hour) according to Australian Bureau of Statistics figures [28]. The costs of productivity loss due to presenteeism were calculated by multiplying the percentage of rating scale indicating the degree of health problem affected productivity while working score× number of hours actually worked per week) × 45 AUD.

#### Secondary outcomes

##### Patient-reported outcomes

Nine patient-reported outcomes were included: (i) The severity of pain and disability were assessed using the VISA-A [29]; (ii) The worst pain level experienced in the last week with an NPRS (11- point scale, 0 = no pain, 10 = worst imaginable pain); (iii) Patient Impression of Change (PIC) which is a 7-point Likert scale including two questions; 1) “How would you describe your Achilles tendon pain now, compared to before you began the treatment?” and 2) “How would you describe your ability to perform physical activities (such as walking, running, housework) now, compared to before you began the treatment?” [30]; (iv) Patient-Acceptable Symptom State instrument [31] which involved a yes or no response to two questions: “Currently are you satisfied with your condition?”, and “Would you recommend this treatment to another person who has Achilles pain?”; (v) Health-related quality of life was measured using the 5-level EQ-5D version (EQ. 5D 5 L index value and overall health state [VAS]); (vi) Physical activity using 7-day Recall Physical Activity Questionnaire [32]; (vii) Fear of movement or re-injury: Kinesiophobia was measured with the Tampa Scale for Kinesiophobia (TSK) [33]; (viii) Pain catastrophising was measured using the Pain Catastrophising Scale (PCS) [34], and (ix) the painDETECT questionnaire for neuropathic pain was only assessed at baseline [35].

##### Plantarflexor strength tests

A custom-built ankle dynamometer (participants seated with 50° knee flexion) was used to assess plantarflexor torque during maximal voluntary isometric contraction, rate of torque development and force matching (see additional file 2 for details). Force matching involved maintaining ankle plantarflexor force equivalent to 10% of their maximal voluntary isometric contraction with visual feedback on a screen 1.5 m in front of them.

### Sample size

We made a pragmatic decision that we would be able to achieve our feasibility aim by recruiting 48 participants to be randomised into one of four factorial arms (*n* = 12 per trial arm as a rule of thumb recommended by Julious) [36].

### Data management and analysis

Entered data were checked for accuracy by two study investigators (FH, PM). Statistical analysis was undertaken on coded data (group allocation concealed). Data from the most painful side were analysed for people with bilateral Achilles pain. SPSS (version 25, IBM Corp., Armonk, NY, USA) was used for statistical analysis. Means and standard deviations were determined for parametric data, medians and interquartile range for non-parametric continuous data, and frequency counts for discrete data. Mean change and standardised response mean (SRM = mean change over time within group/ standard deviation of changes scores over time) from baseline to 6 and 12 weeks with 95% confidence intervals were calculated to provide an estimate of the within group effect size for all secondary outcomes. The SRM magnitude was interpreted (as per Hopkins) as very large when ≥1.2, moderate when ≥0.6, and small when ≥0.2 [37]. The PIC 7-point Likert scale was dichotomised for analyses (“very much improved” and “improved” represents treatment effectiveness).

The maximal voluntary isometric contraction torque data (Nm) were extracted directly from PowerLab (AD Instruments Corp, Dunedin, NZ) whereas the rate of torque development data (Nm/s) and force match data were exported to Excel (Microsoft Corporation, Redmond, WA). The peak rate of torque development (0–50 Nm scale window) was analysed using a custom-written software program (rehabtools.org, Sunshine Coast, Australia). This method used the peak value obtained from an iterative 50 ms averaging window, which has been shown to be highly reliable [38]. Whilst it is a shorter duration than that used in other clinical populations such as people living with stroke [39] this window was shown to be most reliable in our pilot analysis and is less susceptible to a plateau effect from rapid contractions with rise durations shorter than longer windows [39]. The coefficient of variation of torque for the 15 s sampling window was used to represent fluctuations in force-matching (ratio of standard deviation to the mean torque).

Patterns of missing data were analysed using Little’s Missing Completely at Random test [40]. Data substitution was not applied for missing data given this was a feasibility study with a small sample. However, an effort was made to collect the patient-reported outcomes data especially for those who discontinued the intervention.

## Results

**Fig. 2:**
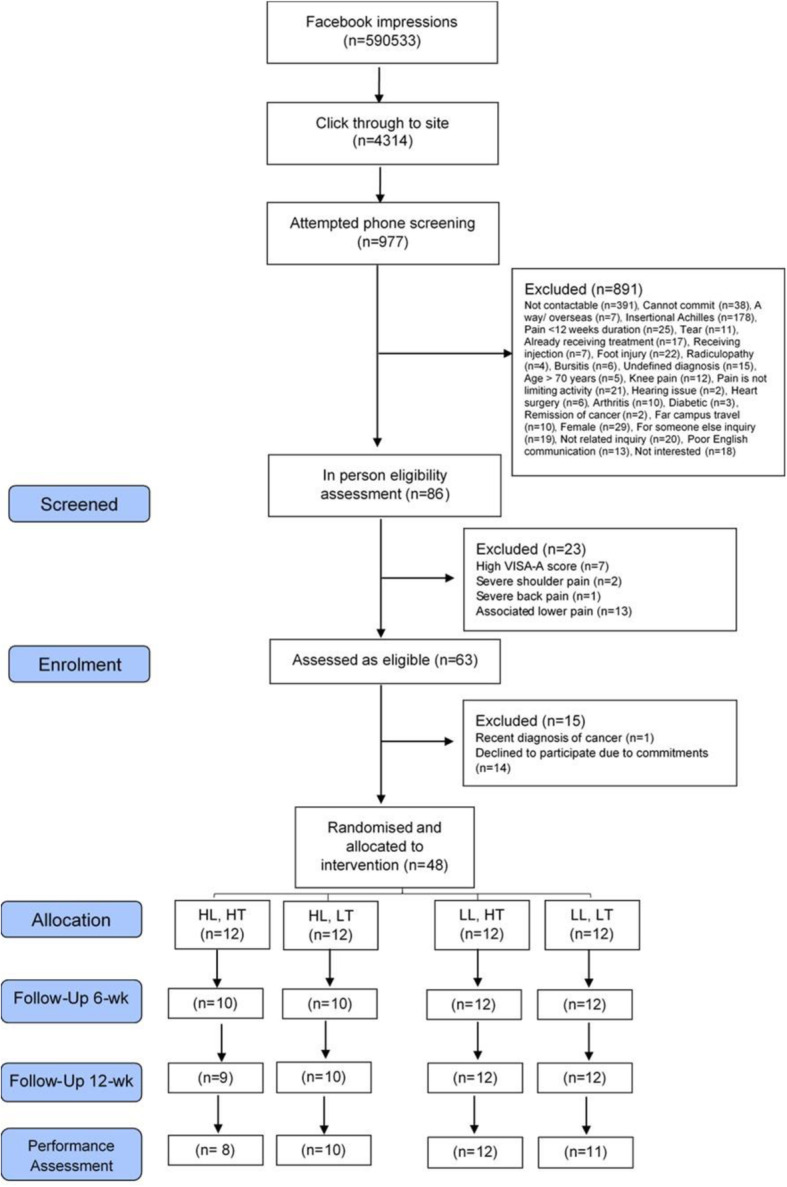
The CONSORT flow diagram of participants through the study

The sample consisted of 48 men, aged 20 to 65 years (mean age 43.2 ± 10.4 years). The duration of symptoms ranged from 3 to 240 months (two outliers of 240 months, median = 24). The BMI ranged from 20 to 45 kg/m^2^. None of the participants reported neuropathic pain (Table 2). From 1043 initial contacts, 86 people were screened in-person, 63 were eligible and 48 consented (Fig. 2).

**Table 2:** Participant characteristics at baseline for each group. Values are mean (SD) unless otherwise noted

	High Load		Low Load	
	with High TUT (*n* = 12)	with Low TUT (*n* = 12)	with High TUT (*n* = 12)	with Low TUT (*n* = 12)
Age, years	42.0 (11.4)	43.0 (11.3)	41.6 (7.2)	46.3 (11.9)
Height, cm	177.8 (6.2)	176.6 (10.3)	178.1 (8.2)	175.0 (8.2)
Mass, kg	89.0 (17.9)	97.0 (18.1)	84.6 (17.1)	94.5 (13.7)
Body mass index, kg/m^2^	28.0 (4.6)	31.1 (5.3)	26.6 (4.4)	30.6 (6.4)
*Employment*^*a*^				
*Full-time*	10 (83)	8 (67)	10 (83)	9 (75)
*Part-time*	0	1 (8)	0	1 (8)
*Casual*	1 (8)	1 (8)	0	1 (8)
*Self-employed*	0	2 (17)	1 (8)	1 (8)
*Student*	1 (8)	0	1 (8)	0
Duration of symptoms, months^b^	11.0 (39)	54.0 (83)	18.0 (30)	12.0 (36)
Dominant side, − right^a^	11 (92)	11 (92)	8 (67)	10 (83)
Presentation, unilateral/ bilateral ^a^	7 (58) / 5 (42)	6 (50) / 6 (50)	4 (33) / 8 (67)	6 (50) / 6 (50)
Achilles tendon AP diameter, mm^c^	6.9 (1.3)	7.8 (1.9)	7.3 (2.2)	8.5 (3.1)
Prior exercise treatment, yes ^a^	3 (25)	4 (33)	6 (50)	8 (67)
Classified Achilles pain quality ^a,d^	7 (58)	10 (83)	9 (75)	9 (75)
*Intensity of Achilles pain* ^a^				
*Mild*	3 (25)	3 (25)	2 (17)	4 (33)
*Moderate*	9 (75)	5 (42)	8 (66)	4 (33)
*Severe*	0	4 (33)	2 (17)	4 (33)
VISA–A	55.3 (13.4)	44.8 (18.1)	46.0 (15.8)	54.7 (9.8)
Pain Detect	10.0 (5.2)	12.2 (6.1)	10.0 (6.0)	10.0 (5.0)

*Abbreviations*: *TUT* time-under-tension, *VISA–A* Victorian Institute of Sports Assessment–Achilles questionnaire. ^a^n (%). ^b^Median (interquartile range). ^c^Measured using b-mode sonography. ^d^Measured using pain mapping app

### Primary outcomes

#### Rate of conversion, recruitment, and retention

**Fig. 3:**
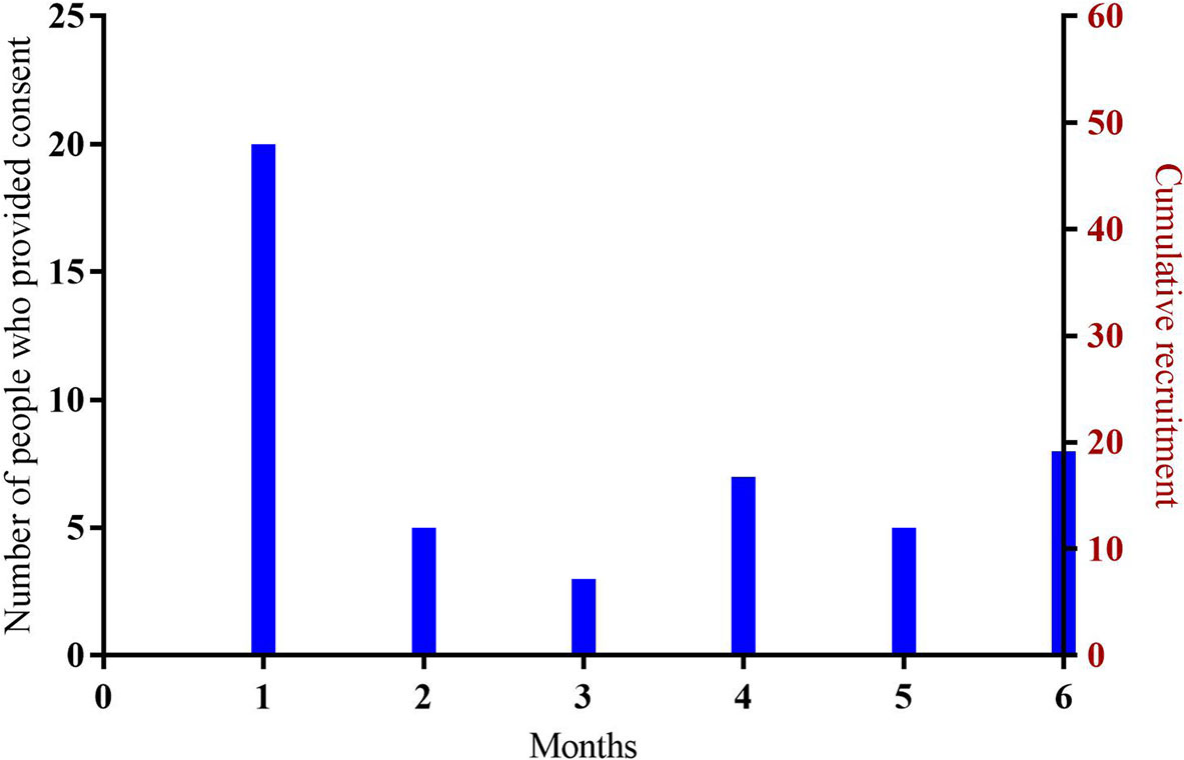
Monthly and cumulative recruitment to the feasibility trial

Both conversion and retention rate were acceptable. The conversion rate was 76% (48/63) (Fig. 2). The recruitment rate ranged from 3 to 20 per month over the 6-months recruitment window (Fig. 3) because the Facebook strategy was intermittent (i.e. stopped for 4 months during holiday periods). Forty-one (85%) participants were recruited via Facebook (total spend $9052.91 AUD; average spend of $221 AUD per person recruited) and the remaining (7 [15%]) via clinical networks and the community.

Five participants did not complete the study (Fig. 2) giving a retention rate of 90% (43/48). Four participants dropped out because of the burden of the intervention, and one participant dropped out due to severe back pain. Between 86 and 98% of outcome data were collected at each timepoint.

#### Exercise adherence and fidelity

**Fig. 4:**
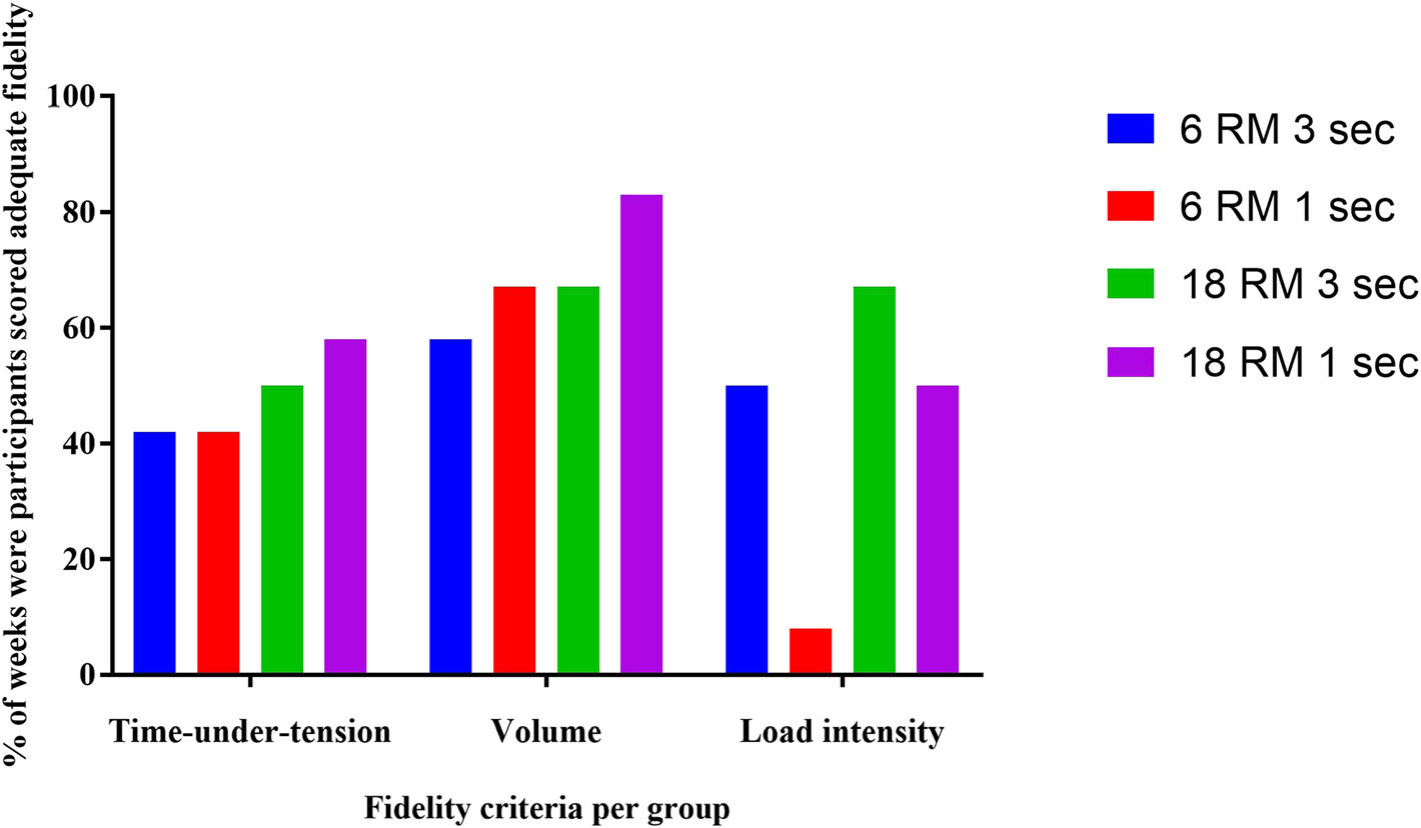
Exercise fidelity of the individual exercise dose parameters for each participant per group

Exercise adherence and fidelity data are presented in Table 3. The proportion of adherence to the supervised sessions was 71 to 92% and total adherence was 49 to 68% (1–2 sessions completed out of 3 per week). Fidelity varied substantially between criteria, with highest fidelity (58 to 83%) seen for the volume criterion. The time-under-tension criterion averaged < 66% across all groups. Fidelity was lower for high-intensity groups (Fig. 4). In general, the success rate (66%) was not reached for both adherence and fidelity outcome.

**Table 3:** Feasibility outcomes. Values are n (%) unless otherwise noted

	High Load		Low Load	
	with High TUT	with Low TUT	with High TUT	with Low TUT
*Session time duration in* minutes^a^	43.4 (12.2)	39.0 (6.1)	53.0 (7.3)	37.1 (7.0)
Adherence^b^				
*Number of zoom sessions completed / 12*	11 (92)	9 (71)	11 (92)	10 (79)
*Number of home sessions completed / 36*	21 (58)	18 (49)	21 (58)	25 (68)
Fidelity of exercise dose parameters in seated				
*Time-under-tension*	5 (42)	5 (42)	6 (50)	7 (58)
*Volume*	7 (58)	8 (67)	8 (67)	10 (83)
*Load intensity*	6 (50)	1 (8)	8 (67)	8 (67)
Fidelity of exercise dose parameters in standing				
*Time-under-tension*	5 (42)	3 (25)	6 (50)	6 (50)
*Volume*	7 (58)	7 (58)	8 (67)	7 (58)
*Load intensity*	5 (42)	4 (33)	9 (75)	7 (58)
No. of participants who reporting adverse event	8 (67)	8 (67)	8 (67)	6 (50)
Severity of adverse events ^c^				
*Mild*	4 (33)	6 (50)	8 (67)	3 (25)
*Moderate*	3 (25)	2 (17)	0	3 (25)
*Severe*	1 (8)	0	0	0
Achilles related adverse events	3 (25)	4 (33)	4 (33)	4 (33)
Severity of Achilles related adverse events				
*Mild*	3 (25)	4 (33)	4 (33)	3 (25)
*Moderate*	0	0	0	0
*Severe*	0	0	0	0
No. of participants who used co-interventions	3 (25)	2 (17)	2 (17)	4 (33)
No. of participants using paracetamol	4 (33)	5 (42)	4 (33)	6 (50)
Total paracetamol tablets used ^d^	16 (33)	31 (33)	33 (25)	17 (25)

*Abbreviations*: *TUT* time-under-tension. ^a^ Mean (SD); ^b^Median (percentage); ^c^ Mild: some discomfort noted but without disruption of daily life that goes within 24–28 h; Moderate: discomfort enough to affect/reduce normal activity that goes within 3-5 days; Severe: complete inability to perform daily activities and lead a normal life and that requires medical intervention; ^d^tablet dose = 500 mg;

#### Incidence of adverse events

There were between six and eight participants per group who experienced an adverse event and a total of 64 reported adverse events over the 3 months (Table 3). Twenty-one participants (70%) reported mild adverse events that included Achilles pain, headache, and pain in other areas [shoulder, wrist, back]). Eight participants (17%) reported moderate adverse events (included back, anterior knee and shin pain) that occurred more frequently in the high-intensity groups (21% versus 13%). There was only one serious adverse event that was not related to the interventions (back pain that required surgery).

#### Use of co-interventions

There were similar rates of co-intervention use between groups (Table 3). Common co-interventions included myotherapy, acupuncture, and topical non-steroidal anti-inflammatory agents (i.e. diclofenac gel). Four to six participants in each group reported using paracetamol for their associated pain, during the 12 weeks of intervention.

Table 3. Feasibility outcomes. Values are n (%) unless otherwise noted.

#### Feasibility of future economic evaluation

Over the three-month study period, the direct cost was 1050 AUD and indirect cost was 37 AUD per participant. This included costs associated with participants who withdrew or missed sessions. The 3 months gym membership cost was 180 AUD per participant. Average presenteeism was valued at 412 AUD per participant over the three-month study period. Only one participant reported absenteeism (8 h) due to their Achilles tendinopathy, which equated to a cost of 765 AUD.

### Secondary outcomes

#### Patient-reported outcomes

**Fig. 5:**
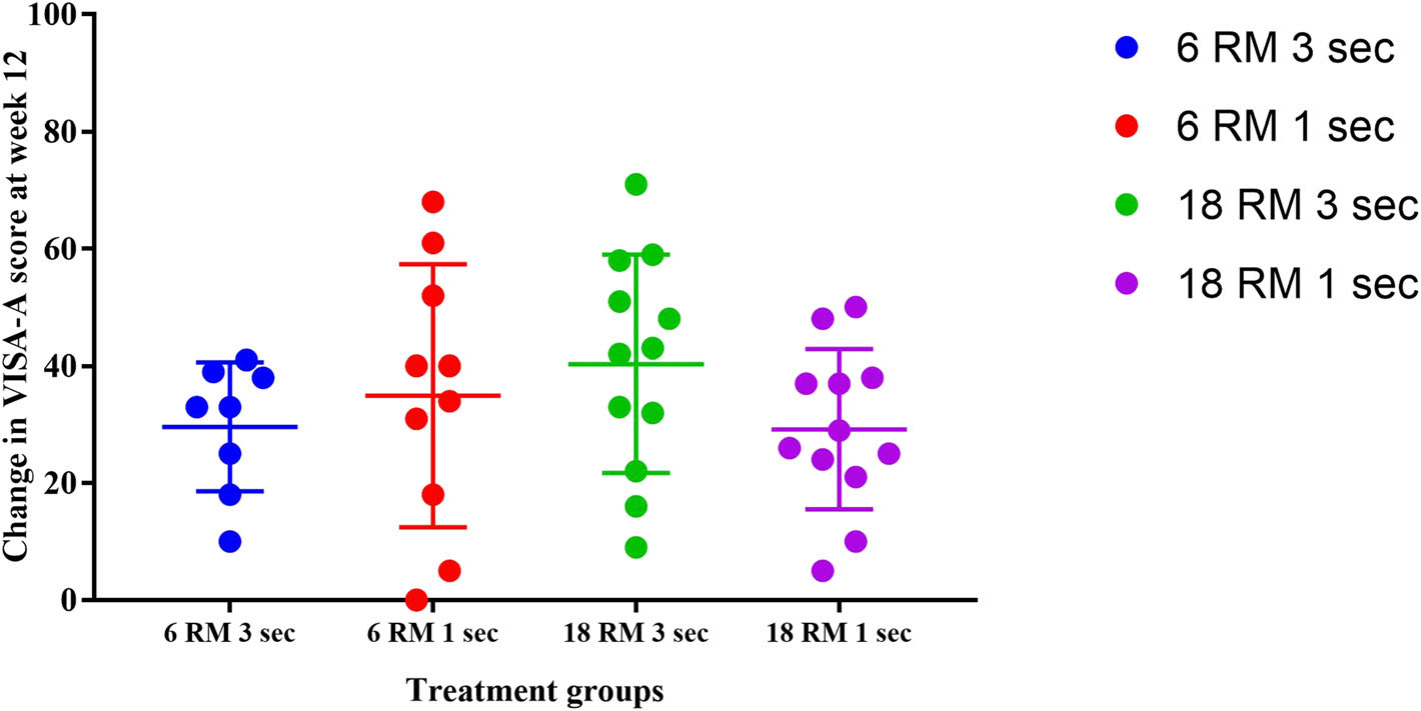
Individual participants changes in VISA-A scores with the intervention

All groups displayed improvement in self-reported outcome measures during the study (Table 4). At 12 weeks, the mean improvement in VISA-A scores was between 26 to 40 points (Fig. 5). The worst pain over the last week decreased by an average of 3 to 4 points. More than 75% of participants reported an improvement in pain and function and satisfaction with condition post-intervention.

**Table 4:** Patient-reported outcomes with change score and standardised response mean. Values are mean (SD) unless otherwise noted

	High Load		Low Load	
	with High TUT	with Low TUT	with High TUT	with Low TUT
Self-reported measures				
Severity of pain using VISA-A ^a^ / 100				
Baseline	55.3 (13.4)	44.8 (18.1)	46.0 (15.8)	54.7 (9.8)
Week-6	69.3 (14.8)	65.2 (16.7)	63.2 (16.8)	68.5 (14.0)
Week-12	81.5 (11.2)	80.7 (17.5)	86.3 (9.0)	83.8 (13.0)
Change score at 6-weeks, [95%CI]	14.0 [11,17]	15.0 [11,19]	17.2 [14,20]	14.0 [11,17]
Change score at 12-weeks, [95%CI]	22.0 [19,25]	22.4 [19,25]	40.3 [37,43]	29.2 [26,32]
SRM baseline-week 6	0.9	0.5	1.3	1.0
SRM baseline-week 12	1.4	0.6	2.2	2.1
Worst pain using Numerical Pain Rating Scale / 10^b^				
Baseline	5.0 (2.2)	4.8 (3.2)	4.7 (3.0)	5.0 (3.0)
Week-6	2 .4 (2.5)	2 .2 (1.0)	3.1 (2.2)	3.3 (2.2)
Week-12	3 .4 (2.7)	2.3 (2.0)	2.1 (2.3)	2.3 (1.8)
Change score at 6-weeks, [95%CI]	−3.0 [−4.-2]	−2.0 [−4.-1]	−2.0 [− 4,1]	−1.1 [− 2.1]
Change score at 12-weeks, [95%CI]	− 2.1 [− 3,1]	−4.0 [−5,-2]	−2.0 [− 5,-0.5]	−2.5 [− 5,-1]
SRM baseline-week 6	− 1.2	− 0.7	− 0.5	− 0.4
SRM baseline-week 12	− 1.1	− 0.9	− 0.8	−0.9
EQ-5D-5L, Index ^c^/ 1				
Baseline	0.71 (0.10)	0.71 (0.17)	0.72 (0.10)	0.74 (0.10)
Week-6	0.79 (0.09)	0.76 (0.16)	0.76 (0.10)	0.78 (0.14)
Week-12	0.84 (0.11)	0.88 (0.14)	0.84 (0.11)	0.87 (0.12)
Change score at 6-weeks, [95%CI]	0.08 [0.1,0.3]	0.04 [−0.2,0.3]	0.02 [− 0.12,0]	0.03 [− 0.2,3]
Change score at 12-weeks, [95%CI]	0.14 [0.1,0.5]	0.2 [− 0.1,0.3]	0.12 [− 0.1,0.3]	0.13 [0,0.3]
SRM baseline-week 6	0.8	0.2	0.2	0.3
SRM baseline-week 12	1.1	1.0	1.1	1.1
EQ-5D-5L, VAS ^d^ / 100				
Baseline	67.8 (15.2)	70.2 (17.8)	72.8 (16.6)	69.6 (12.0)
Week-6	82.2 (8.5)	75.3 (9.4)	80.0 (8.3)	75.7 (13.6)
Week-12	83.4 (13.2)	82.6 (15.5)	82.3 (10.3)	74.0 (11.5)
Change score at 6-weeks, [95%CI]	15.0 [12,18]	6.4 [5,8]	7.0 [4,10]	6.0 [4,8]
Change score at 12-weeks, [95%CI]	19.0 [16,22]	14.0 [12,14]	9.0 [7,11]	4.0 [2,6]
SRM baseline-week 6	0.9	0.6	0.4	0.8
SRM baseline-week 12	0.9	2.0	1.0	0.4
7-Day Activity Recall ^e^				
Baseline	1041 (170)	1322 (257)	1017 (200)	1303 (296)
Week-6	1071 (241)	1311 (237)	997 (194)	1229 (256)
Week-12	837 (555)	1314 (211)	1046 (175)	1275 (285)
Change score at 6-weeks, [95%CI]	29.3 [19,40]	−11.0 [−23,2]	−20.0 [−29,-11]	74.0 [− 62,-86]
Change score at 12-weeks, [95%CI]	86.2 [82,96]	−3.0 [− 15,5]	29.0 [− 29,-10]	− 28 [−62,-86]
SRM baseline-week 6	0.1	0	0	0
SRM baseline-week 12	0.3	0	0.2	0
Tampa Scale for Kinesiophobia ^f^/ 68				
Baseline	39.8 (8.7)	39.7 (3.6)	37.2 (4.9)	35.1 (12.5)
Week-6	34.5 (8.3)	35.0 (6.1)	34.9 (4.1)	30.8 (6.8)
Week-12	30.2 (6.4)	29.3 (6.1)	32.3 (6.4)	27.2 (4.5)
Change score at 6-weeks, [95%CI]	−5.2[−8,-2]	−5.0[−7,-3]	−2.3[−4,1]	−4.3[−7,-2]
Change score at 12-weeks, [95%CI]	−12.1[−14,-10]	−10.4[−12,-9]	− 5.0[−7,-3]	− 8.0[− 11,-5]
SRM baseline-week 6	−0.6	−0.6	−0.7	−0.5
SRM baseline-week 12	− 2.0	− 1.4	−0.6	−0.6
Pain Catastrophising Scale ^g^/ 52				
Baseline	12.8 (6.4)	17.3 (16.5)	9.4 (7.7)	11.4 (11.3)
Week-6	6.9 (3.2)	8.0 (7.0)	7.3 (5.9)	6.1 (5.4)
Week-12	4.3 (4.2)	5.6 (6.2)	5.3 (5.9)	2.7 (2.1)
Change score at 6-weeks, [95%CI]	−6.0[−8,-4]	−10.2[− 13,-7]	−2.2[−4,-1]	−5.3[− 8,-3]
Change score at 12-weeks, [95%CI]	−8.0[− 10,-7]	−13.0[− 16,-10]	− 4.2[− 6,-2]	−9.0[− 11,-6]
SRM baseline-week 6	−1.2	− 0.7	− 0.3	− 0.5
SRM baseline-week 12	−1.3	− 0.7	− 0.5	− 0.8
Patient Impression of Change for pain ^h,i^				
Week-6 (improve)	8 (67)	9 (82)	5 (42)	8 (67)
Week-12 (improve)	7 (78)	10 (100)	10 (91)	9 (82)
Patient Impression of Change for function ^h,i^				
Week-6 (improve)	7 (58)	8 (73)	4 (33)	9 (75)
Week-12 (improve)	7 (78)	10 (100)	10 (91)	9 (100)
Patient-Acceptable Symptom State instrument for satisfaction ^i^				
Week-6 (50% improve)	5 (42)	1 (10)	4 (33)	5 (42)
Week-6 (100% improve)	7 (58)	9 (90)	8 (67)	7 (58)
Week-12 (50% improve)	2 (22)	0	2 (18)	2 (17)
Week-12 (100% improve)	7 (78)	10 (100)	9 (82)	10 (83)

*Abbreviations*: ^a^Scores on the VISA–A range from 0 (worst Achilles symptoms) to 100 (no Achilles tendinopathy). ^b^ worst pain during the week scored on the NPRS range from 0 (no pain) to 10 (worst pain imaginable). ^c^Scores on the EQ-5D-5L Index Value range from < 0 (worse than dead) to 0 (dead) to 1 (full health). ^d^Scores on the EQ-5D-5L VAS range from 0 (worst imaginable health state) to 100 (best imaginable health state). ^e^Scores on the PAR are represented as daily energy expenditure (kilocalories per day). ^f^ Tampa score ranges from 17 (no fear of movement) to 68 (greater fear of movement). ^g^ Scores on CPS range from 0 (no pain) to 52 (higher levels of pain catastrophising). ^h^ dichotomised scores to “improved” and “not improved”. ^i^n (%)

#### Plantarflexor strength tests

**Fig. 6:**
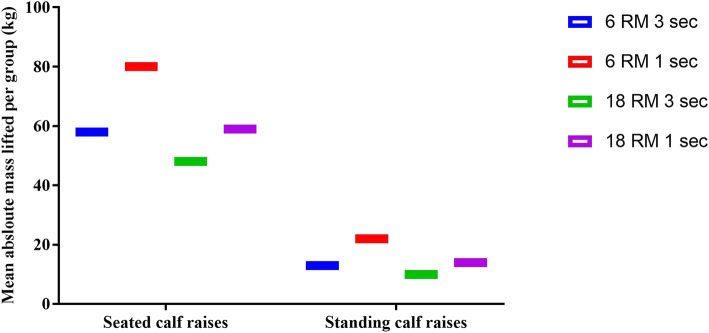
Overall absolute mass lifted during the intervention

All groups displayed improvement in all strength tests (Table 5). The magnitude of mass lifted increased by about 25% in seated and 10% in the standing positions (Fig. 6).

**Table 5:** Performance outcomes with change score and standardised response mean (mean ± SD)

	High Load		Low Load	
	with High TUT	with Low TUT	with High TUT	with Low TUT
Ankle plantarflexion torque (Nm)				
Baseline	188.4 (43.1)	194.9 (60.8)	188.6 (53.8)	188.5 (66.2)
Week-12	225.8 (32.9)	220.0 (42.8)	204.9 (58.0)	206.8 (40.0)
Change score, [95%CI]	48.3 [44,53]	27.0 [22,32]	16.3 [12,21]	24.4 [19,30]
SRM baseline-week 12	1.4	0.8	1.0	0.6
Ankle plantarflexion rated of torque development (Nm/s)				
Baseline	644.5 (235.3)	637.4 (326.6)	635.9 (215.8)	557.1 (197.5)
Week-12	850.4 (174.4)	738.9 (189.0)	699.9 (183.4)	668.6 (170.4)
Change score, [95%CI]	232.0 [220,244]	134.0 [122,146]	64.0 [54,74]	153.0 [143,163]
SRM baseline-week 12	0.8	0.6	0.6	0.9
Plantarflexor coefficient of variation of torque				
Baseline	1.9 (0.8)	1.9 (0.4)	2.3 (1.0)	2.2 (1.0)
Week-12	1.6 (0.2)	1.6 (0.9)	1.9 (1.0)	1.7 (1.0)
Change score, [95%CI]	−0.7 [−0.1,0]	−0.3 [−0.8,0.3]	−0.5 [−1.2,0.3]	−0.5 [−1.3,0.4]
SRM baseline-week 12	−0.9	−0.3	−0.6	−0.3
Mass lifted in seated (kg)				
Baseline	54.5 (20.2)	38.3 (23.4)	45.8 (10.2)	56.3 (17.2)
Week-12	77.5 (41.8)	64.0 (19.1)	70.8 (25.0)	79.4 (26.0)
Change score, [95%CI]	15.0 [11,18]	28.0 [24,31]	37.0 [34,39]	24.2 [21,28]
SRM baseline-week 12	0.5	1.3	2.2	1.2
Mass lifted in standing (kg)				
Baseline	13.1 (8.7)	11.2 (14.0)	9.5 (7.1)	8.3 (9.5)
Week-12	27.4 (14.1)	18.0 (12.0)	16.2 (11.4)	22.1 (23.3)
Change score, [95%CI]	14.0 [11, 16]	9.3 [−7, 12]	3.3 [1,6]	12.0 [9,14]
SRM baseline-week 12	1.0	1.0	0.2	0.7

## Discussion

This pilot randomised trial has demonstrated that the interventions are safe (only one unrelated serious adverse event), and the rate of conversion, recruitment, and retention were acceptable. Adherence to the gym-based intervention was acceptable for the weekly telehealth supervised sessions but not for the two non-supervised sessions. Exercise fidelity varied substantially between criteria and was unsatisfactory for the time-under-tension criterion across all groups. Prior to progressing to a fully powered trial, further work is necessary to identify and trial strategies to improve exercise adherence and fidelity. The major cost appeared to be related to provision of the intervention. Unlike other chronic pain conditions [41], the amount of time lost from work and productivity loss at work related to this condition was minimal, which raises the question whether economic evaluation is warranted in a fully powered trial.

Retention was 79% across the groups that exercised at high-intensity, compared to 100% in the low-intensity groups. We do not know whether this difference in dropout rate is related to the exercise interventions (e.g. people undertaking high load-intensity found it more difficult and were less likely to continue). There was a higher rate of moderate adverse events, based on inspection of estimates not significance testing, in the high-intensity (5/24 = 21%) versus low-intensity groups (3/24 = 13%). Follow-up qualitative interviews did not identify any themes that may explain this observation (unpublished data). We took precautions to reduce muscle soreness from exercise (revising down exercise intensity for all groups initially).

Only one serious adverse event was reported during the study. A participant in the high load-intensity with lower time-under-tension group developed severe low back pain that required surgery. This adverse event did not occur whilst the participant was undertaking the exercise prescribed as part of this trial, and it is not possible to ascertain whether the prescribed exercise was a factor in this occurrence. Mild and anticipated Achilles related adverse events such as ankle sprain and tendon pain provocation were common and occurred with similar frequency in each group (25 to 33%). It appears that overall, the interventions described in this study are safe.

Our interventions have similar characteristics to the ‘heavy slow resistance training’ intervention utilised by Beyer et al. [42]. Heavy slow resistance involves three sessions of gym-based calf loading exercise per week. There were, however, differences in reported exercise adherence between our pilot trial (49 to 68%) and the Beyer et al. trial (92% for heavy slow resistance). Discrepant adherence rates may be explained by methodological differences between the trials. First, Beyer et al. prescribed three 2-legged exercises (three exercises in total) which would have been less time consuming than the four exercise (2 on each limb) in our study. Second, the socio-geographical environment (Copenhagen versus Melbourne) may have influenced adherence (e.g. commute time to the gym or traffic congestion favouring Copenhagen). Third, the adherence data was recorded more frequently in our study (weekly by our physiotherapists from patient report during the telehealth sessions versus a single supervised session plus patient diaries in the Beyer study) which may have influenced accuracy by reduced recall bias [42]. Future studies could benefit from better understanding barriers to patients’ exercise adherence, in order to identify and implement strategies to improve adherence across all trial groups, such as more supervised exercise sessions, either synchronously or asynchronously (e.g. via patients self-recording exercise sessions).

Exercise fidelity was important for ensuring that the exercise groups varied in the exercise parameters that were being compared including volume, intensity and time-under-tension. There was varied fidelity rate across all the trial groups with the lowest fidelity seen for the time-under-tension criterion (25 to 42%). Adequate time-under-tension was achieved if calf raise tempo was judged by the telehealth rater (physiotherapist) to be in time with metronome (auditory cue) during the telehealth sessions. Errors related to poor internet connection and delayed or freezing video that may impact the rating, were reduced by re-rating any trials in which this was perceived by the rater. This criterion may have been too stringent because the overall time-under-tension may have been satisfactory even if there were timing violations for some of the repetitions. In future trials comparing different levels of time-under-tension, we recommend comparing total time-under-tension per set. Although they did not assess time-under-tension, Sancho et al. recently reported exercise (calf raises and hopping) fidelity ranging from 22 to 64% for volume and load-intensity [43].

High-intensity groups had lower fidelity compared to low-intensity groups. This could be related to training until volitional failure. Utilising a repetition in reserve paradigm may be more appropriate [44] and can be considered in a future trial. Fidelity strategies may need to be targeted towards the high-intensity groups as they seem to have more difficulty performing the required load-intensity. This could be a practical issue i.e. participants lifting heavy weight, or fear issues.

At 12 weeks, there was an improvement in patient-reported pain and function scores measured with the VISA-A questionnaire, ranging from 26 to 40 points. In a recent systematic review of 31 studies among individuals with Achilles tendinopathy undergoing a calf muscle loading program mean (SD) change in patient-reported pain and function (measured using the VISA-A questionnaire) was 21.1 (6.6) points [10]. Our findings are one to three standard deviations above this pooled mean. A clinically meaningful change is suggested to be 10-points [42, 45, 46] and this is also the Cochrane collaboration recommended minimal clinically important difference for a 100-point function scale [47]. However, our study did not include a control group, so the influence of natural history or placebo is not known. Other secondary outcomes also changed favourably in all groups. The improvements were likely to be clinically meaningful as the estimated effect size were moderate to large ≥0.6. Although our three-month outcome is appropriate for our efficacy trial, longer term follow-up would eventually be needed to determine the long-term effects of our interventions.

Variability of the VISA-A outcome in our data can be used to estimate the sample size for a future fully powered randomised controlled trial. Ninety-four participants (i.e. 47 per group) would provide power of over 80% to detect an effect size of 10-points on the VISA-A questionnaire [42, 45, 46] with the significance level set at *p* < 0.05. A pooled standard deviation of 17.2 was derived from the 4 arms in this pilot study. The sample size calculation assumes that intention to treat analysis is being applied so ignores non-adherence and drop-out.

This study has limitations. First, limiting recruitment to men reduces generalisability. This was justified in our trial because the exercise parameters being tested may have a differential effect on tissue adaptation and clinical outcomes between sexes [22]. Another limitation related to including only men is that our feasibility and pilot data (including the sample size estimate) may not extrapolate to women and this needs to be considered carefully in planning a fully powered trial. Second, although the outcome assessor was blinded to treatment allocation, participants and physiotherapists providing care via videoconference were not, so there is the risk of performance and detection bias. This was mitigated partly by expressing uncertainty to participants regarding potential comparative efficacy between groups. Preferences of participants or physiotherapists towards certain exercise parameters may still be an issue and we recommend assessing outcome expectations, or implementation of procedures to blind participants (and assessors) in future trials. Third, we recruited a majority of participants via social media and it is not certain whether they represent the range of individuals presenting for Achilles tendinopathy management in primary care. However, the age and severity profile (VISA-A scores) is similar to other studies also undertaken in Australia among individuals with Achilles tendinopathy [48].

## Conclusions

The results of this study suggest that high and low-intensity and time-under-tension loading protocols are feasible and safe for individuals with mid-portion Achilles tendinopathy. Future trials should consider strategies to optimise exercise adherence and fidelity.

## Supplementary Information

**Additional file 1.** Load progression.

**Additional file 2.** Ankle plantarflexor strength testing.

## Data Availability

The datasets used and/or analysed during the current study are available from the corresponding author on reasonable request.

## Abbreviations

RM: Repetition maximum
VISA-A: Victorian Institute of Sports Assessment – Achilles questionnaire
MVIC: Maximal Voluntary Isometric Contraction
RTD: Rate of Torque Development
NPRS: Numerical Pain Rating Scale
PIC: Patient Impression of Change
WPAI: Work Productivity and Activity Impairment Questionnaire
