# Supplementary material for: LOAD-intensity and time-under-tension of exercises for men who have Achilles tendinopathy (the LOADIT trial): a randomised feasibility trial
Source: BMC Sports Sci Med Rehabil. 2021 May 25;13:57. doi: 10.1186/s13102-021-00279-z (PMC8152048; doi:10.1186/s13102-021-00279-z)
Supplement: Supplementary file 1 — Additional file 1. Load progression. [file 13102_2021_279_MOESM1_ESM.docx]

# **Additional file 1:** Load progression

The load was based on the volitional muscular failure, where failure is defined as an inability to do another full repetition. The instruction to participants was that they should feel that they cannot do any more repetitions when they complete the set. A range of ± one repetition was allowed. *Load was influenced by the self-reported pain during the exercise, with < 5 out of 10 on numerical rating scale (where 10 is the worst pain imaginable)*

| 0 | 1 | 2 | 3 | 4 | 5 | 6 | 7 | 8 | 9 | 10 |
| --- | --- | --- | --- | --- | --- | --- | --- | --- | --- | --- |
|  |  |  |  |  |  |  |  |  |  |  |

**No pain Worst pain**

Safe zone= acceptable pain Risky zone= not acceptable pain

| **Exercise level** | **Specificities** | **Progression** | **Progression condition** |
| --- | --- | --- | --- |
| Isometric calf raises | 5 sets x Body weight in standing and unloaded in seated x 45 seconds contraction at the highest load that is tolerable | Progress to isotonic calf raises | If unable to load or pain ≥ 5/10 |
| Double calf raises on flat ground | 4 sets x 6 or 18 RM x 6- or 2-seconds contraction | Progress calf raises over a weight plate | If minimal load is acceptable while ankle in neutral position and pain severity is within an acceptable range |
| Double calf raises over a weight plate | 4 sets x 6 or 18 RM x 6- or 2-seconds contraction | Progress single calf raises | if the unilateral position is too painful |
| Single calf raises on flat ground | 4 sets x 6 or 18 RM x 6- or 2-seconds contraction | Progress calf raises over a weight plate | If minimal tendon load is acceptable while ankle in neutral position and pain severity is within an acceptable range |
| Single calf raises over a weight plate | 4 sets x 6 or 18 RM x 6- or 2-seconds contraction | Progress adding weight | If tendon load is acceptable from the full range plantarflexed position and pain severity is within an acceptable range |
