# Supplementary material for: LOAD-intensity and time-under-tension of exercises for men who have Achilles tendinopathy (the LOADIT trial): a randomised feasibility trial
Source: BMC Sports Sci Med Rehabil. 2021 May 25;13:57. doi: 10.1186/s13102-021-00279-z (PMC8152048; doi:10.1186/s13102-021-00279-z)
Supplement: Supplementary file 2 — Additional file 2. Ankle plantarflexor strength testing. [file 13102_2021_279_MOESM2_ESM.docx]

# **Additional file 2:** Ankle plantarflexor strength testing

A custom-built ankle dynamometer was used for testing. Participants were seated barefoot in the dynamometer with the ankle in plantargrade and knee joint flexed to 50° to optimise the activation of soleus and gastrocnemius. A standardised warm-up procedure consisted of five minutes of static bicycling followed by four familiarisation trials with two seconds of MVIC. Two actual trials were recorded, and additional trials were repeated until the MVIC achieved of the two highest trials were within 5% to determine a valid and repeatable result. The highest reading was used for subsequent analysis. Participants were instructed to push as hard and fast as possible. For force-matching, participants did two familiarisation trials of submaximal effort at target torque levels of 10% of MVIC with 60 seconds rests between trials and 90 seconds rest between the tasks. Participants were asked to maintain an ankle plantarflexor force equivalent to 10% of their MVIC with visual feedback on a screen 1.5 meters in front of them (line on the screen indicates the target force). They were instructed to gradually reach the required force level and then maintain this force for 15 seconds. This force-matching task results in fluctuation around the target output, with smaller fluctuation indicating better muscle force control.
